# Effects of RAC1 on Proliferation of Hen Ovarian Prehierarchical Follicle Granulosa Cells

**DOI:** 10.3390/ani10091589

**Published:** 2020-09-06

**Authors:** Thobela Louis Tyasi, Xue Sun, Xuesong Shan, Simushi Liswaniso, Ignatius Musenge Chimbaka, Ning Qin, Rifu Xu

**Affiliations:** 1 Department of Animal Genetics, Breeding and Reproduction, College of Animal Science and Technology, Jilin Agricultural University, Changchun 130118, China; louis.tyasi@ul.ac.za (T.L.T.); xuesun1128@163.com (X.S.); xsshan@gmail.com (X.S.); smliswaniso@gmail.com (S.L.); ignatius.chimbaka@gmail.com (I.M.C.); 2 Joint Laboratory of Modern Agricultural Technology International Cooperation, Ministry of Education, Jilin Agricultural University, Changchun 130118, China

**Keywords:** chicken, RAC1, granulosa cell, cell proliferation, steroidogenesis

## Abstract

**Simple Summary:**

The growth and development of ovary follicles is an intricate, highly organized process involving many local intra-ovarian factors. Ras-related C3 botulinum toxin substrate1 (RAC1) is speculated to be associated with prehierarchical follicle development of hen ovaries. The current study initially revealed *RAC1* mRNA to be expressed in varied-size follicles and stroma and its expression levels in the prehierarchical follicles of 1.0–3.9 mm, 6.0–6.9 mm and 7.0–8.0 mm in diameter were remarkably higher than the other groups. Moreover, RAC1 protein was mainly expressed in the oocytes and granulosa cells (GC), as well as in stromal tissues of the follicles. To understand the exact roles of the *RAC1* gene in regulation of follicular GC proliferation and differentiation, siRNA interference and overexpression of the *RAC1* gene were conducted. Our experiments demonstrated that the *RAC1* gene can significantly promote the expression of mRNA and proteins of *FSHR*, *CCND2*, *CYP11A1*, *PCNA* and *StAR* genes in GC and directly elevate the proliferation of GC in vitro. These results indicated RAC1 played a crucial role in regulation of GC proliferation and differentiation and steroidogenesis during the development of prehierarchical follicles. This study provided a base for elucidating the molecular mechanisms underlying the biological effect of RAC1 on the hen ovary follicle growth and development.

**Abstract:**

RAC1 belongs to the small G protein Rho subfamily and is implicated in regulating gene expression, cell proliferation and differentiation in mammals and humans; nevertheless, the function of RAC1 in growth and development of hen ovarian follicles is still unclear. This study sought to understand the biological effects of RAC1 on granulosa cell (GC) proliferation and differentiation of hen ovarian prehierarchical follicles. Firstly, our results showed expression levels of *RAC1* mRNA in the follicles with diameters of 7.0–8.0 mm, 6.0–6.9 mm and 1.0–3.9 mm were greater than other follicles (*p* < 0.05). The RAC1 protein was mainly expressed in oocyte and its around GCs and stromal tissues of the prehierarchical follicles by immunohistochemistry. Further investigation revealed the *RAC1* gene remarkably enhanced the mRNA and protein expression levels of FSHR (a marker of follicle selection), CCND2 (a marker of cell-cycle progression and GC differentiation), PCNA (a marker of GC proliferation), StAR and CYP11A1 (markers of GC differentiation and steroidogenesis) (*p* < 0.05). Furthermore, our data demonstrated siRNA interference of RAC1 significantly reduced GC proliferation (*p* < 0.05), while *RAC1* gene overexpression enhanced GC proliferation in vitro (*p* < 0.05). Collectively, this study provided new evidence that the biological effects of RAC1 on GC proliferation, differentiation and steroidogenesis of chicken ovary follicles.

## 1. Introduction

Egg production is one of the major important economic traits of chickens. Efficient egg production primarily depends on the regulation of ovarian prehierarchical follicle recruitment, follicular selection and differentiation and preovulatory follicular hierarchy and ovulation. Follicle selection and differentiation are important steps that determine if prehierarchical follicles develop into preovulatory follicles [1]. Only one percent of the prehierarchical follicles selected during selection and differentiation (6.0–8.0 mm in diameter) eventually mature and is ovulated [2]. During the follicle selection stage, RAC1 was recently identified to exert a great influence on the development of prehierarchical follicles [3]. The small GTP-binding protein, ras-related C3 botulinum toxin substrate1 (RAC1) is a member of the small G protein Rho subfamily [4]. RAC1 has a GTPase binding region, at which cycling between an active GTP-bound state and an inactive GDP-bound state acts as molecular switches in the body [5]. Moreover, RAC1 serves as an important signaling molecule, which is associated with the regulation of gene expression, mitosis, proliferation, apoptosis and angiogenesis. Recent studies exhibited RAC1 mediates MAPK and Src/AKT/erk1/2 signaling pathways [6,7,8]. Additionally, RAC1 also is implicated in the regulation of numerous events of reproduction that include embryo implantation, fixing in mammalian oocytes, meiotic spindle stability and human embryonic epithelial morphogenesis [9,10,11,12]. Compelling evidence suggested that RAC1 protein was expressed in both human ovaries and chicken follicles and regulated the formation of primary mouse follicles by promoting transcription of GDF9 and BMP15 [13]. Accordingly, we predicted Rac1 is required for follicular selection and differentiation; however, the role and its molecular mechanism of RAC1 in granulosa cell (GC) proliferation and differentiation of hen ovarian follicles remains poorly understood.

During the process of chicken follicular predominance selection, differentiation and hierarchy, *FSHR*, *CCND2*, *PCNA*, *StAR* and *CYP11A1* genes play crucial roles. The expression of *FSHR* mRNA in GC of candidate predominant follicles increases significantly, which causes them to further proliferate and differentiate to produce progesterone [14,15]. Furthermore, the expression of Cyclin D2 (CCND2) is induced by follicle-stimulating hormone (FSH) and CCND2 acts as a marker of cell-cycle progression and cell differentiation [16,17]. Proliferating cell nuclear antigen (PCNA) is related to DNA replication machinery and regulates cell proliferation; therefore, it can act as an indicator of cell proliferation [18,19]. Together, the expression of *CCND2* and *PCNA* contribute to cell differentiation and proliferation.

Cytochrome P450 family 11 subfamily A member 1 (CYP11A1) and steroidogenic acute regulatory protein (StAR) regulate steroid formation at the time of the development and maturation of chicken ovarian follicles. StAR moves cholesterol molecules to the mitochondrial inner membrane where they are transformed to pregnenolone by the action of CYP11A1, promoting GC differentiation indirectly [20,21]. The importance of these genes were demonstrated when steroidogenic incompetency in differentiating GCs of prehierarchical follicles was primarily due to a lack of StAR and CYP11A [22,23]. Currently, it still remains unclear how RAC1 regulates the expression and functions of *FSHR*, *CCND2*, *PCNA*, *StAR* and *CYP11A1* in GC. In the current study, we examined the expression and localization of RAC1 in chicken ovarian follicles and the effect of interference or overexpression RAC1 on GC *FSHR*, *CCND2*, *PCNA*, *StAR* and *CYP11A1* mRNA expression in GC. Moreover, direct regulation of RAC1 in GC proliferation was revealed in vitro. The biological roles of Rac1 in GC proliferation, differentiation and steroidogenesis of ovarian prehierarchical follicles were initially determined in the chicken.

## 2. Materials and Methods

### 2.1. Chickens

All procedures were carried out according to the Institutional Animal Care and Use Committee (IACUC) of Jilin Agricultural University (IACUC) [Changchun, China; Permission No. GR (J) 19-89]. Hy-Line Brown layers originating from the same feeding environment and nutrition level were selected as experimental subjects for the experiments conducted at the Animal Science and Technology College of Jilin Agricultural University. The hen was kept in single cage, freely accessing to feed and water and exposed to a 16L: 8D photoperiod. At the age of 21 weeks, twenty layers were obtained from the flock and euthanized; the ovary from every hen was promptly evacuated and set into 0.9% ice-cold NaCl solution. The classifications of hen ovarian follicles were based on a previous study [2], prehierarchical follicles (1.0–3.9 mm, 4.0–4.9 mm, 5.0–5.9 mm, 6.0–6.9 mm, 7.0–8.0 mm in diameter) and hierarchical follicles F6, F5, F4, F3, F2 and F1 (9.0–40 mm in diameter) respectively [24,25]. A representative segment of every ovary was gotten and promptly solidified in fluid nitrogen and stored at −80 °C.

### 2.2. Primary Culture of Chicken Ovarian GC

According to the previously published methods the primary culture of GCs from prehierarchical follicles measuring 6.0–8.0 mm in diameter was performed. Briefly, following granulosa layers were immediately isolated from the prehierarchical follicles (6.0–8.0 mm in diameter). After washing with M199 medium (Gibco, New York, NY, USA), granulosa layer cells were dispersed in 0.2% collagenase (type 2; Sangon, Shanghai, China) for 30 min and enhanced with 10% fetal calf serum (Gibco, New York, NY, USA) in humidified chambers at 37 °C, 5% CO_2_ [2,26,27]. Cultured GC used in experiments were purified and quantified. The specificity of the GC was determined by H & E staining and immunofluorescence [24].

### 2.3. Quantitative Real-Time Polymerase and Chain Reaction (RT-qPCR)

To assess mRNA expression of focus genes in follicles and GC, RT-qPCR was used as previously described [24]. Primer 5 (Premier Biosoft International, Palo Alto, CA, USA) was performed to design the primers while their synthesis was carried out by the company of TaKaRa (Japan; Table 1). In this study, an internal control gene *18S rRNA* was applied in each reaction system. Each PCR reaction was replicated thrice in an ABI 7500 Real-Time PCR system (Applied Biosystems, Foster City, CA, USA) and the PrimeScript™ RT-PCR Kit (Takara, Japan) was used according to the manufacturer’s directions. The 2^−ΔΔCt^ way was exploited to analyze the relative expression of the target genes. The PCR procedure were as follows—each PCR program was started at 95 °C for 120 s, proceeded by incubation at 95 °C for 10 s; then, there were 40 cycles of denaturation at 95 °C for 15 s and with a final extension at 65 °C for 1 min. PCR efficiencies were identified utilizing a relative standard curve consequential from a diluted cDNA reaction mixture (a 2-fold dilution series with five measuring points). All standard curves had R2 values ranging from 0.997 to 0.999. The PCR effectiveness was between 90% and 110% (Table 1).

### 2.4. Immunohistochemistry Assay

The localization of the RAC1 protein was determined using immunohistochemistry assay in ovarian prehierarchical follicles. The methods were the same as previously described with a slight modification [24]. Briefly, chicken follicles were harvested and conserved with 4% glutaraldehyde-polyoxymethylene solution immediately, dehydrated and paraffin embedding according to routine methods. Paraffin sections were immersed in the distilled water for 5 min, phosphate buffered saline (PBS) was rinsed three times and endogenous peroxidase ablation was blocked by 3% peroxide-methanol at room temperature. Deparaffinized sections were incubated with rabbit anti-cRAC1 (1:50, Abcam, Cambridge, MA, USA) at 4 °C overnight, washed three times with PBS, followed by incubation with goat anti-rabbit secondary antibody (1:100, Abcam, Cambridge, MA, USA) at room temperature for 60 min with a horseradish peroxidase (HRP) labeled (Table 2). Negative control sections were performed with omission of the primary antibody and replaced with the same concentration of normal rabbit serum. No specific staining was observed in these controls. Photomicrographs were taken with a biological microscope (JNOEC XS-213, Optics and Electronics Co., Ltd. Nanjing, China).

### 2.5. Construction of Recombinant Expression Vector and Cell Transfection

Chicken *RAC1* cDNA sequences (NM_205017.1) were amplified by primers as follows: forward: 5′-GGATCCCGCGAATGCATCTAGATATC-3′ and reverse: 5′-CTCGAGGGCGTAATCATGGTCATAGC-3′ (*Xho* I and *BamH* I linker sequences are underlined). The mature *RAC1* gene was cloned into the *Xho* I and *BamH* I sites of the vectors pUC57-simple by the company of Biobuffer Biotech Service (Wuhan, China). Next the PUC57-*RAC1* recombined plasmids were released upon digestion with restriction enzyme *Xho* I and *BamH* I (TaKaRa, Japan) and ligated into the expression vector pYr-adshuttle-4 expressive vector (Biobuffer Biotech Service, Wuhan, China). As we have previously reported, *RAC1* gene expression was transfected with recombinant plasmid vector pYr-adshuttle-4-*RAC1* [21]. More specifically, the randomly grouped GCs (6.0–8.0 mm) were transfected by a pYr-adshuttle-4 blank vector and reconstructed plasmid pYr-adshuttle-4-*RAC1* using Lipofectamine (lip) 2000 transfection reagent (Invitrogen, Carlsbad, CA, USA). Next, at a density of 1 × 10^5^ cells/well in a 24-well plate, cultures were done in a medium that had 1μL/mL Polybrene (hexadimethrine bromide, Sigma). These later incubated with 5% CO_2_ at 37 °C. After being continually cultured for 24 h, GCs were collected and lysed and RT-qPCR analyses post continues cultures in 24 h [1].

### 2.6. Western Blotting

The western blotting was performed using total cellular extracts as previously described [3]. In brief, an equal amount of protein in dropping conditions was separated using approximately 10% (*w/v*) SDS-Polyacrylamide gel and was electro-transferred to a Protran nitrocellulose membrane. The sections were blocked in 5% BSA blocking solution for 1 h and incubated at 4 °C overnight. All primary antibodies and diluted concentration are showed in Table 2. Following the sections were washed with scrubbing solution for four times and incubated for 30 min at room temperature with a secondary antibody (Table 2).

### 2.7. Transfection of siRNA

The precise siRNA sequences of targeting *RAC1* gene was designed by Invitrogen siRNA Wizard V3.1 software, which is available on website (http://www.sirnawizard.com/design_advanced.php). Blast was performed on mRNA chicken genome database to remove cross-silence phenomenon with non-target gene. The most efficient *RAC1* siRNA, 5′-CCCACAGUCUUUGACAACUTT-3′ was verified by RT-qPCR and Western blotting analysis. Furthermore, the siRNA negative control was the scrambled siRNA, 5′-UUCUCCGAACGUGUCACGUTT-3′, which is a non-targeting gene. In accordance with the manufacturer’s directions, *RAC1* and control siRNAs were transfected strictly using the Lip 2000 (Invitrogen, Carlsbad, CA, USA) until GCs (6.0–8.0 mm) were grown to 70% confluence (48 h).

### 2.8. Cell Proliferation Analysis

5′-Ethynyl-2′-deoxyuridine (EdU) incorporation assay was implemented to evaluate cell proliferation viability using the Cell-LightTM EdU imaging kit (C10310-1, RiboBio, Guangzhou, China) according to the manufacturer’s instructions. In brief, transfected and control cells were inoculated and cultured in 96-well flat-bottom plates at a density of 1 × 10^5^ cells/well and incubated in a CO_2_-incubator at 37 °C for 24 h. At room temperature, cells were exposed to 50 nM of EdU for 2 h at 37 °C and fixed with 4% paraformaldehyde for 15 mi. Then the sections were rinsed twice with glycine (2 mg/mL) for 5 min, incubated with 100 uL of 0.5% Trion X-100 for 10 min, reacted with 100 uL of 1× Apollo reaction cocktail for 30 min, followed by 1 × Hoechst 33342 (200 mL per well) for staining nuclei. The fluorescent microscope (Olympus, Tokyo, Japan) was employed to image the stained cells. Twenty fields were analyzed and averaged. Each experiment was done in threes with five replicates [3].

### 2.9. Statistical Analysis

Several batches of sampled hens were used in this experiment at last thrice. The SPSS 17.0 statistical software (IBM, Armonk, NY, USA) was performed for all the statistical analyses. Quantification of mRNA expression of target genes were analyzed by RT-qPCR. Four independent products were amplified from each hen. The Kolmogorov-Smirnov test was used to check if the quantitative data satisfied the requirement of normality. A one-way ANOVA followed by a Dunnett Multiple Comparison test after confirming the data had a normal distribution. All data are presented as means ± SEM, *p* < 0.01 or *p* < 0.05 was taken to be statistically significant.

## 3. Results

### 3.1. Expression of RAC1 in Chicken Ovarian Follicles

**Figure 1:**
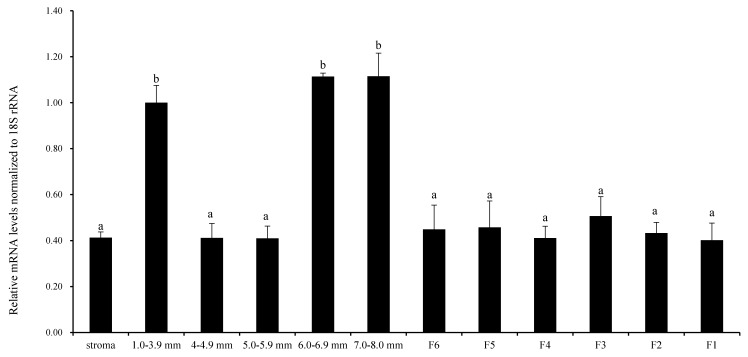
Quantification of *RAC1* mRNA expression by RT-qPCR analysis in different-sized ovary follicles. It was normalized with respect to *18S rRNA*. The stroma from the ovary contains matrix tissue from large follicles (≥1 mm in diameter), small follicles (<1 mm in diameter), atretic follicles, somatic and other cells. Represented are means and standard error of means (mean ± SEM) of data from chickens (*n* = 10). Values with unlike superscripts designate a statistically significant differences (*p* < 0.05).

Figure 1 shows that the relative expression level of *RAC1* mRNA was examined in undifferentiated prehierarchical follicles (1.0–8.0 mm in diameter) and preovulatory follicles (F6-F1). The expression of *RAC1* mRNA was higher in follicles with diameters of 7.0–8.0 mm, 6.0–6.9 mm and 1.0–3.9 mm than other sizes and all grade follicles (*p* < 0.05). No noteworthy contrast was seen between follicles of various sizes and all graded follicles (*p* > 0.05). These results preliminarily revealed that *RAC1* gene takes part in chicken follicular growth and development regulation at mRNA transcription level.

### 3.2. Localization of RAC1 in the Chicken Ovarian Follicles

**Figure 2:**
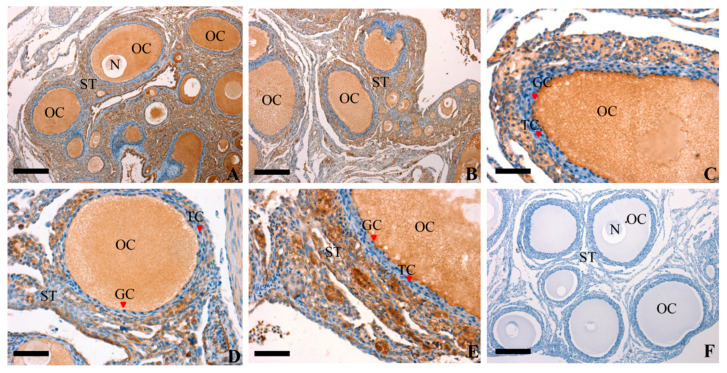
Representative photomicrographs of RAC1 protein immunolocalization (in brown) in the prehierarchical follicle of ovarian. Panel (**A**,**B**), oocytes and GCs were strongly stained within the different size of prehierarchical follicles; Panel (**C**,**D**), a larger developing prehierarchical follicles, which contains two or three layers of GCs; Panel (**E**), a larger prehierarchical follicles and more layers of GCs and thecal cells developing in this stage; Panel (**F**), RAC1 negative controls, which were treated with pre-immune serum and no specific staining was observed. Oocyte (OC), granulosa cell (GC), theca cell (TC), stroma (ST) and nucleus (N) are indicated. Scale bar = 200 μm (**A**,**B**,**F**); 100 μm (**C**,**D**,**E**).

Immunohistochemistry was used to study the localization of RAC1 protein at every developmental stage. As show in Figure 2, an unequivocally positive brown staining for RAC1 expression was detected in the oocytes (OC) and GC within the follicles of different sizes and the stromal tissues (ST) neighboring the follicles. Besides, a weak positive brown staining was discovered in thecal cells (TC). The current study demonstrated that the RAC1 protein was not only predominantly present in the OC, GC and ST but also in the TC. These findings showed RAC1 protein could play an imperative role in regulating the progress and development of the ovarian follicles through paracrine/autocrine ways within the follicles.

### 3.3. RAC1 Promoted the GCs Proliferation

**Figure 3:**
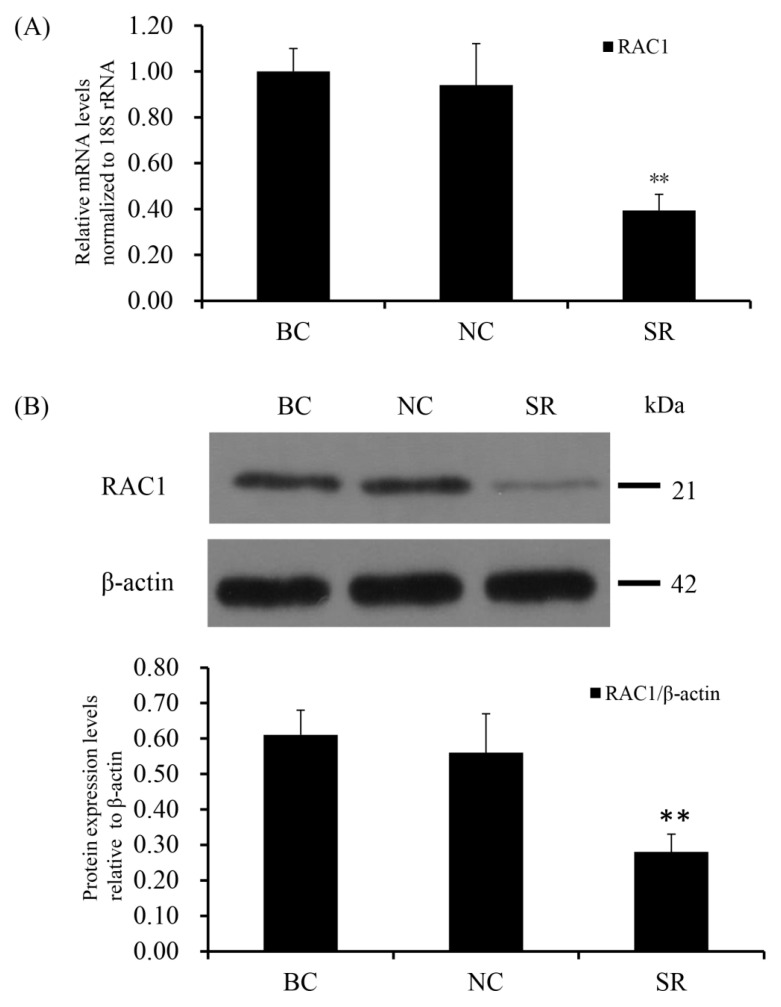
*RAC1* mRNA and protein expression levels after cells transfected with *RAC1*-specific siRNA. Different treatment groups were transfected into GCs from the prehierarchical follicles. SR group: exact siRNA *RAC1*; NC group: scrambled siRNA; BC group: absence of siRNA. (**A**) *RAC1* mRNA expression was scrutinized by RT-qPCR analysis with *18S rRNA* as a normalization gene. (**B**) RAC1 protein expression was detected by western blotting analysis against β-actin protein. Data represent means ± SEM (*n* = 10). Bars with superscripts suggest the statistically noteworthy difference in comparison to the control groups (** *p* ˂ 0.01).

**Figure 4:**
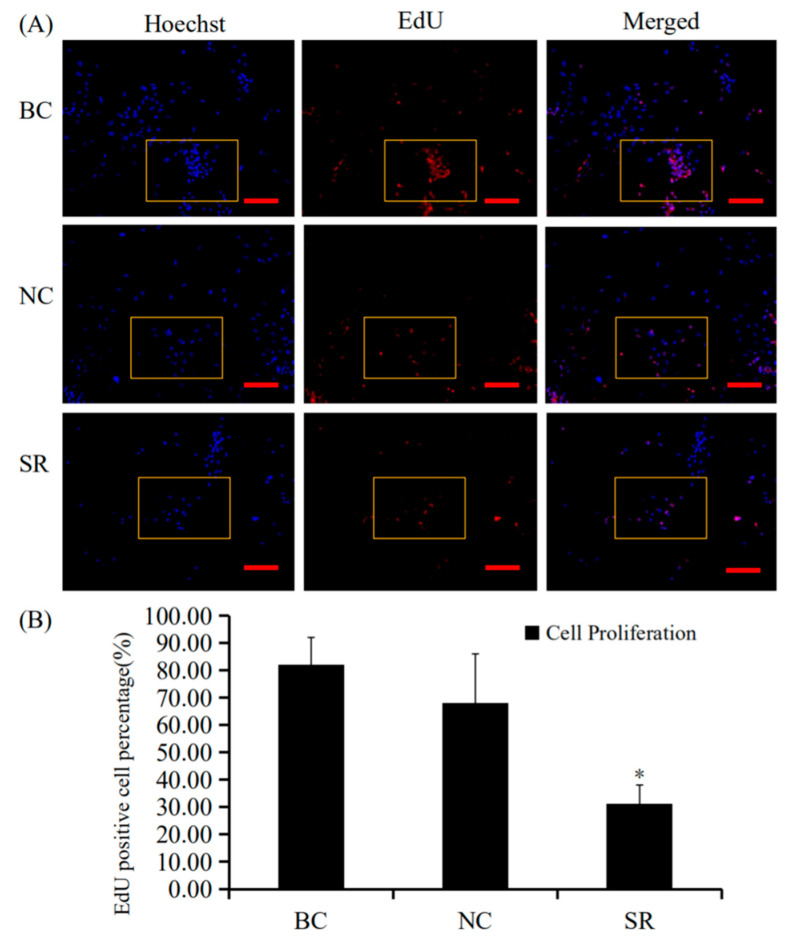
Effects of silencing RAC1 on granulosa cell proliferation. The effects of silencing *RAC1* on the proliferation of GCs were studied by EdU cell proliferation assay. (**A**) The GCs were transfected with different treatment groups. SR group: exact siRNA *RAC1*; NC group: scrambled siRNA; BC group: absence of siRNA. All nuclei of cells showed a blue fluorescence suggestive of Hoechst33342 staining. The red fluorescence showed in EdU-labeled cells suggesting the newly produced DNA (original magnification ×20) (Figure S1, original magnification ×40). (**B**) The proliferation percentage of GC post cell transfected with the *RAC1* specific siRNA. Data represent means ± SEM (*n* = 10). Bars with superscripts suggest the statistically noteworthy difference in comparison to the control groups (* *p* < 0.05).

SiRNA-mediated *RAC1* gene silencing in the GCs of prehierarchical follicles was done to detect the biological role of *RAC1* gene. The effect of RAC1 on the viability of GCs was detected by use of EdU cell proliferation assays. RT-qPCR and Western blotting were used to check the efficiency of knocking down *RAC1* in cells transfected with *RAC1*-specific siRNA (*p* < 0.01; Figure 3). As can be seen in Figure 4, the proliferation of GC decreased after interference of *RAC1* gene contrasted with the negative control (*p* < 0.05). This result indicates the down-regulation of *RAC1* gene mRNA expression would reduce the stimulation of GC proliferation and inhibit the growth and development of prehierarchical follicles.

### 3.4. Conformity of the Positive Effect of RAC1 on Granulosa Cell Proliferation

**Figure 5:**
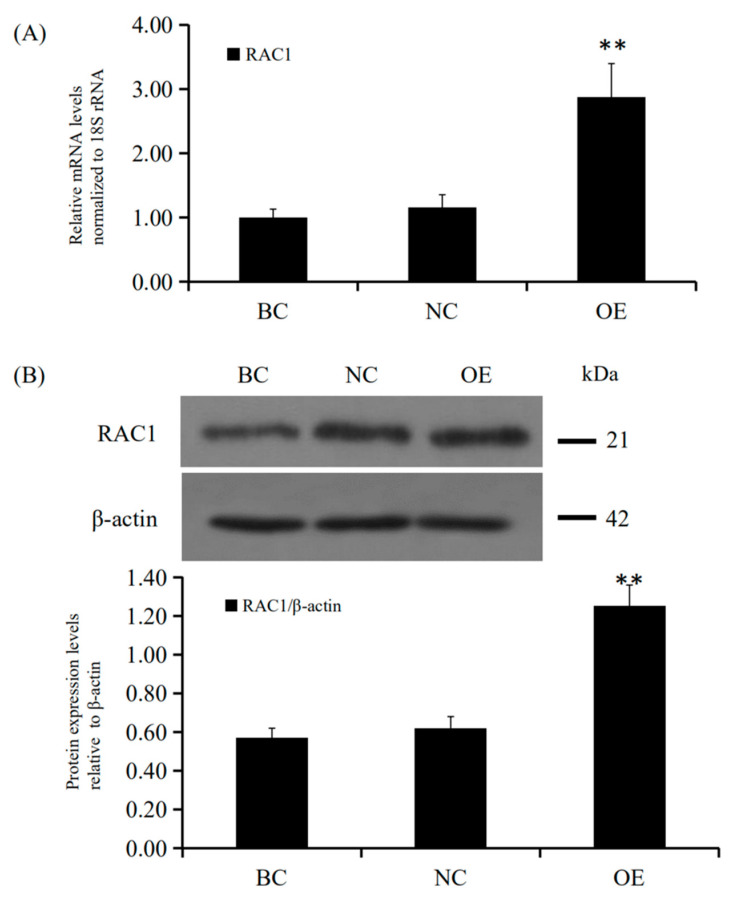
Effects of RAC1 overexpression on GC *RAC1* mRNA and protein expression. Different treatment groups were transfected into GCs from the prehierarchical follicles. OE group: pYr-adshuttle-4-*RAC1* vector; NC group: pYr-adshuttle-4 empty vector; BC group: absence of expression vector. (**A**) *RAC1* mRNA expression was scrutinized by RT-qPCR analysis with *18S rRNA* as a normalization gene. (**B**) RAC1 protein expression was detected by western blotting analysis against β-actin protein. Data represent means ± SEM (*n* = 10). Bars with superscripts suggest the statistically noteworthy difference in comparison to the control groups (** *p* ˂ 0.01).

**Figure 6:**
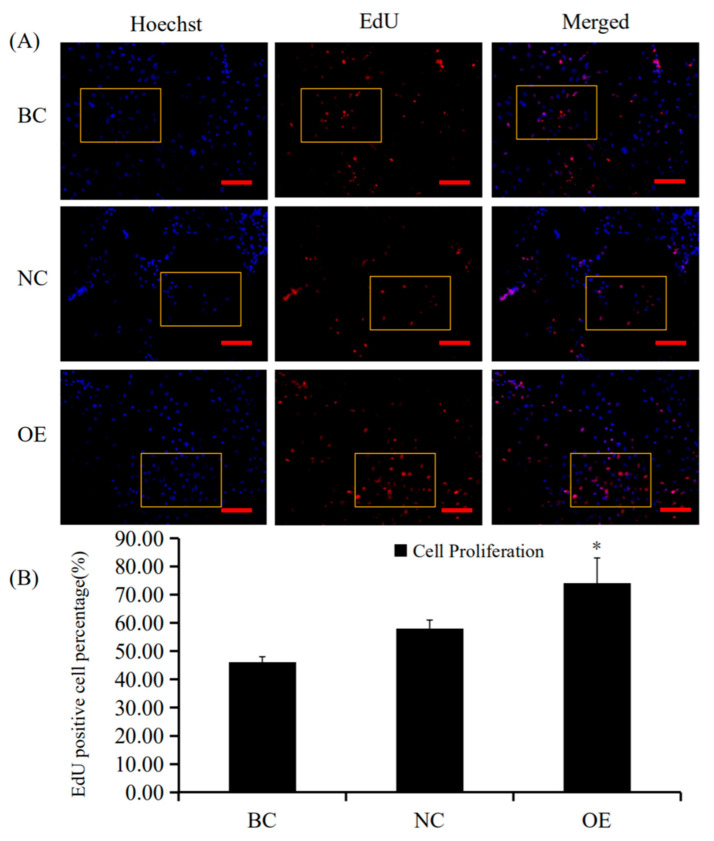
Effects of overexpressing RAC1 on granulosa cell proliferation. The effects EdU cell proliferation assay was used to assess the effects of overexpressing *RAC1* on GC proliferation. (**A**) The GCs were transfected with different treatment groups. OE group: pYr-adshuttle-4-*RAC1* vector; NC group: pYr-adshuttle-4 empty vector; BC group: absence of expression vector. Blue fluorescence was seen in all cell nuclei since Hoechst33342 staining, cells labeled with red fluorescence suggesting their newly produced DNA (original magnification ×20) (Figure S2, original magnification ×40). (**B**) The percentage of GC proliferation after cells were transfected with pYr-adshuttle-4-*RAC1* vector. Data represent means ± SEM (*n* = 10). Bars with superscripts suggest the statistically noteworthy difference in comparison to the control groups (* *p* < 0.05).

Since RAC1 promoted GC proliferation, we further examined the positive effect of the GC *RAC1* gene overexpression. Firstly, the recombinant plasmid vector pYr-adshuttle-4-*RAC1* was transfected into the GC. Secondly, the levels of expression for the *RAC1* mRNA and its protein were determined by RT-qPCR and Western blotting. Twenty-four hours after transfection of the expression vector, *RAC1* mRNA and protein expression were noticeably elevated (*p* < 0.01; Figure 5). Lastly, the proliferation of GC was detected by an EdU assay. The data showed that *RAC1* gene overexpression increased the number of GC (*p* < 0.05; Figure 6). Therefore, these findings revealed that *RAC1* gene might involve in promoting the proliferation of granulosa cells in vitro.

### 3.5. RAC1 Stimulated the mRNA and Protein Expression of FSHR, CCND2, PCNA, StAR and CYP11A1

**Figure 7:**
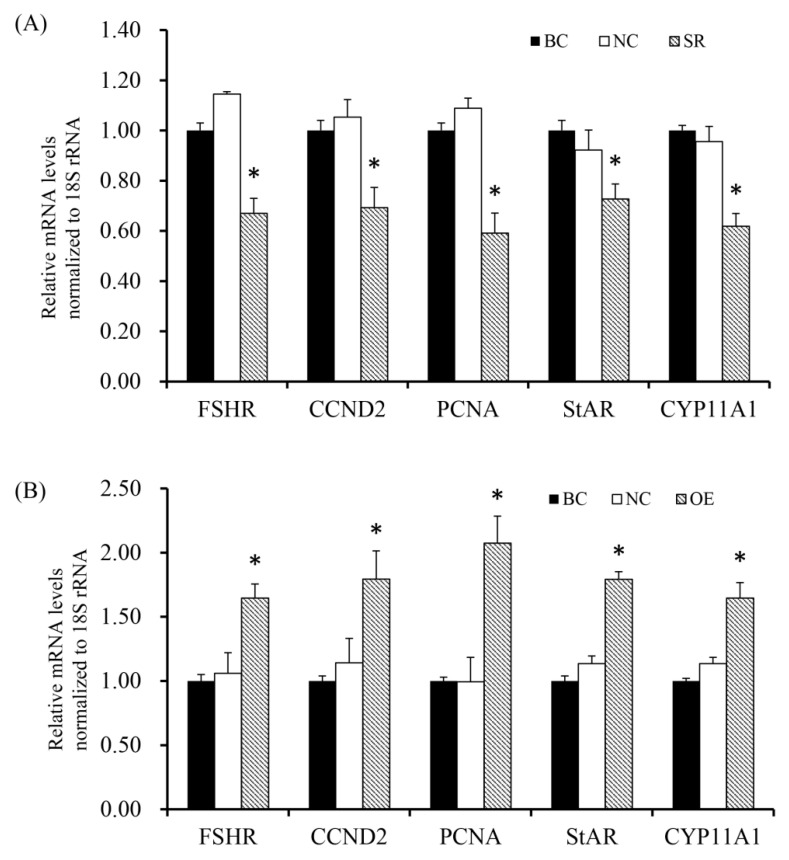
Rac1 stimulates mRNA expression of *FSHR*, *CCND2*, *PCNA*, *StAR* and *CYP11A1* genes. (**A**) Different treatment groups were transfected into GCs from the prehierarchical follicles.SR group: exact siRNA *RAC1*; NC group: scrambled siRNA; BC group: absence of siRNA. Transfection knocked down *RAC1* on *FSHR*, *CCND2*, *PCNA*, *StAR* and *CYP11A1* genes mRNA expression. (**B**) Different treatment groups were transfected into GCs from the prehierarchical follicles. OE group: pYr-adshuttle-4-*RAC1* vector; NC group: pYr-adshuttle-4 empty vector; BC group: absence of expression vector. Transfection overexpressed *RAC1* on *FSHR*, *CCND2*, *PCNA*, *StAR* and *CYP11A1* mRNA expression. Data represent means ± SEM (*n* = 10). Bars with superscripts suggest the statistically noteworthy difference in comparison to the control groups (* *p* < 0.05).

**Figure 8:**
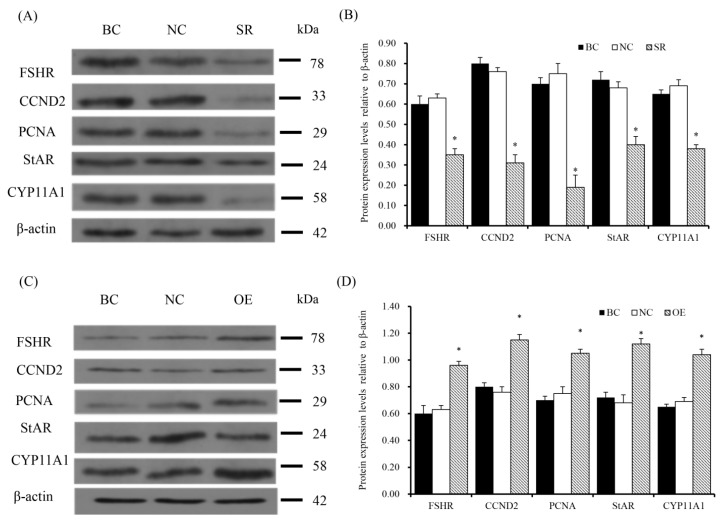
Rac1 prompts protein expression of FSHR, CCND2, PCNA, StAR and CYP11A1. (**A**,**B**) Expression of FSHR, CCND2, PCNA, StAR and CYP11A1 proteins after RAC1 specific siRNA transfection in GC. The loading control used was β-actin. The gels were run and all the blots were cropped under the same experimental conditions. (**C**,**D**) Expression of FSHR, CCND2, PCNA, StAR and CYP11A1 proteins after pYr-adshuttle-4-*RAC1* vector transfection in GC. β-actin was used as the loading control. Data represent means ± SEM (*n* = 10). Bars with superscripts suggest the statistically noteworthy difference in comparison to the control groups (* *p* < 0.05).

To determine effect of RAC1 on the expression of FSHR, CCND2, PCNA, StAR and CYP11A1, RT-qPCR analysis and Western Blotting were performed to detect the expression of essential factors. As shown in Figure 7 and Figure 8, the findings showed mRNA and protein expression levels of FSHR, CCND2, PCNA, StAR and CYP11A1 were down-regulated (*p* < 0.05) after GC were transfected with RAC1-specific siRNA. On the contrary, under incitement of the over manifested RAC1, a sharp increase of *FSHR*, *CCND2*, *PCNA*, *StAR* and *CYP11A1* mRNA and protein expression was examined (*p* < 0.05).

## 4. Discussion

RAC1, a member of the small molecule Rho GTPase family, can switch between the active form of GTP binding and the inactive form of GDP binding. Active RAC1 was originally identified to play a crucial role in regulating cell morphology, cell adhesion and migration [4,5,11]. Recent study showed RAC1 was involved in regulating many reproductive events [28]. In order to understand the role of RAC1 in chicken ovary follicle development, the expression of *RAC1* mRNA and its localization were measured by RT-qPCR and immunohistochemistry assays in ovarian follicles. Our results showed the expression of *RAC1* mRNA was across all the various sized follicles sampled and expressed greater in follicles with diameters of 1.0–3.9 mm, 6.0–6.9 mm and 7.0–8.0 mm. Well known, some follicles are selected to undergo a complex hierarchical regulation in the stage of prehierarchical follicles (6.0–8.0 mm in diameter). The selected follicles eventually mature and ovulate with GC proliferation, differentiation and oocyte development and maturation [25,29]. Our data documenting the expression of *RAC1* mRNA indicates RAC1 may be essential for prehierarchical follicular selection and differentiation. Furthermore, RAC1 protein was not only predominantly expressed in GC and OC but also in TC and ST. Proliferation of GC is an important event of hen follicle selection [30]. Therefore, results suggested that RAC1 may be closely related to granulosa cell proliferation and differentiation via an intra-follicular paracrine or autocrine manner.

GCs produce estradiol and progesterone as a reaction the inducement by gonadotropins (FSH and LH) respectively [31] and interacted with oocytes during follicular development and promoted oocyte development through nutrient exchange and information exchange [32]. In present investigation, RAC1-specific siRNA silencing was performed by transfection into GC of prehierarchical follicles and the outcomes indicated the proliferation levels of GC decreased. To further confirm the results, the recombinant plasmid vector pYr-adshuttle-4-*RAC1* was transfected into the GCs and it showed that *RAC1* gene overexpression increased the number of GC. Previous study has also demonstrated the role of RAC1 in follicular development. RAC1 was verified to be expressed in mouse GC. Additionally, in the same study, silencing RAC1 activity significantly inhibited GC proliferation and promoted its apoptosis [33]. The results directly supported our findings that RAC1 plays a role in promoting GC proliferation. Moreover, RAC1 as the downstream effector of RHOG, mediated signal transmission in GC but the exact regulation pathway is unknown [34]. Our previous study indicated that Slit/Robo GTPase activating proteins (srGAPs) inhibited the activity of the endogenous GTP-CDC42 protein by SLIT2 overexpression; however, no changes in RAC1 activity levels were detected. This suggests the inhibitory impact of SLIT2-ROBO1/2 on GC development and differentiation might be not intervened by the inactivation of GTP-RAC1 [3]. The present results indicated RAC1 was implicated in different size chicken ovary follicles development and acted as a stimulator in granulosa cell proliferation; however, the role of RAC1 to regulate differentiation of granulosa cells and steroidogenesis remains unclear.

To further understand the regulations of RAC1 in GC differentiation, steroidogenesis and follicle selection, as well as cell proliferation, the expressions of FSHR, CCND2, PCNA, StAR and CYP11A1 were detected during overexpression and siRNA interference of *RAC1* expression. As a previous study reported, in follicles with a diameter of 6.0–8.0 mm, undifferentiated GC began to acquire FSH responsiveness and FSHR-mediated cAMP production, which initiated differentiation and selection of dominant follicles [26]. The increased or decreased expression levels of FSHR, under the regulation of RAC1, showed that RAC1 is convoluted in the process of prehierarchical follicle selection and initiating GC differentiation. Furthermore, GC differentiation coincides with increased secretion of StAR, CYP11A1 and CCND2. As previously reported, StAR plays a key role in controlling the rate-limiting step of steroidogenesis and cholesterol access to CYP11A1 [22]. The *CYP11A1* gene encodes the cytochrome P450 cholesterol side-chain cleavage enzyme (P450scc) and this enzyme accelerates the creation of cholesterol into progesterone [24]. To activate synthesis of steroid hormones, CYP11A1 acts as a speed limiting and regulatory agent in this pathway. Accordingly, StAR and CYP11A1 serve as indicators of granulosa cell differentiation and steroidogenesis [17,35,36]. CCND2 mainly controls cell cycle and promotes cell differentiation and serves as a marker of GC differentiation as well as cell-cycle progression [37,38]. The expression of the *PCNA* gene is strongly linked with cell proliferation, which has caused researchers to designate it as a promising biomarker of GC proliferation [19,39,40]. In this study, results showed that siRNA interference of RAC1 significantly reduced the mRNA and protein expression level of StAR, CYP11A1, CCND2 and PCNA in the GCs. The present data demonstrated that RAC1 may play a crucial role in the regulation of GC differentiation and steroidogenesis, as well as cell proliferation. This finding was additionally upheld by the results of RAC1 overexpression. Collectively, these data verified *RAC1* gene plays a pivotal role in regulation of GC differentiation and steroidogenesis as well as GC proliferation, differentiation and follicle selection during the ovarian prehierarchical follicular development and growth in chicken.

## 5. Conclusions

In summary, the current study is the first to reveal that RAC1 is expressed in prehierarchical follicles of chicken ovarian and is largely located in OC and GC. Most of all, it convincingly demonstrated that RAC1 plays a positive role in GC proliferation, differentiation and steroidogenesis of the follicles. These findings contributed to the understanding of a new effect of RAC1 on hen ovary prehierarchical follicle growth and development.

## Figures and Tables

**Table 1:** List of primers used for Quantitative Real-Time Polymerase and Chain Reaction (RT-qPCR).

Gene	Primer Sequences (5′-3′)	Accession No.	Size	Efficiency	Annealing Temperatures
*RAC1*	F: GACCCAAACTTGATTCCTAG	NM_205017.1	232 bp	101.6%	58 °C
	R: GACGGTGCTGTAGGTAAA				
*FSHR*	F: TCCTGTGCTAACCCTTTCCTCTA	NM_205079.1	207 bp	102.3%	60 °C
	R: AACCAGTGAATAAATAGTCCCATC				
*CCND2*	F: AACTTGCTCTACGACGACC	NM_204213.1	150 bp	99.8%	59.5 °C
	R: TTCACAGACCTCCAACATC				
*PCNA*	F: TGAATGAGCCAGTCCAG	NM_204170.2	144 bp	100.5%	59 °C
	R: AGTGTCCCATATCAGCAA				
*StAR*	F: AGCAGATGGGCGACTGGAAC	AF220436.1	147 bp	98.9%	59.5 °C
	R: GGGAGCACCGAACACTCACAA				
*CYP11A1*	F: TCCGCTTTGCCTTGGAGTCTGTG	NM_001001756.1	112 bp	103.1%	59.5 °C
	R: ATGAGGGTGACGGCGTCGATGAA				
*18SrRNA*	F: TAGTTGGTGGAGCGATTTGTCT	AF173612.1	169 bp	102.6%	60 °C
	R: CGGACATCTAAGGGCATCACA				

**Table 2:** Western blotting antibodies.

Protein Target	Primary Antibody	Diluted	Secondary Antibody	Diluted
RAC1	Rabbit anti-cRAC1 (Abcam, Cambridge, MA, USA)	1:1000	anti-rabbit IgG	1:2000
FSHR	Mouse anti-cFSHR (Boster, Biological Technology, Wuhan, China)	1:1000	anti-mouse IgG	1:2000
CCND2	Mouse anti-cCCND2 (Invitrogen, Carlsbad, CA, USA)	1:1000	anti-mouse IgG	1:2000
PCNA	Rabbit anti-cPCNA (Sangon, Biotech Co., Ltd., Shanghai, China)	1:1000	anti-rabbit IgG	1:2000
StAR	Mouse anti-cStAR (Invitrogen, Carlsbad, CA, USA)	1:1000	anti-mouse IgG	1:2000
CYP11A1	Mouse anti-cCYP11A1 (Invitrogen, Carlsbad, CA, USA)	1:1000	anti-mouse IgG	1:2000
β-actin	Mouse anti-cβ-actin (Boster, Biological Technology, Wuhan, China)	1:1000	anti-mouse IgG	1:2000

Antibody concentration: 100 uL, 1 μg/uL. The source of the protein/peptide targets of all the primary antibodies were derived from chicken.

## Supplementary Materials

The following are available online at https://www.mdpi.com/2076-2615/10/9/1589/s1, Figure S1: Effects of silencing RAC1 on granulosa cell proliferation (original magnification ×40), Figure S2: Effects of overexperssing RAC1 on granulosa cell proliferation (original magnification ×40).
