# Supplementary material for: Effects of RAC1 on Proliferation of Hen Ovarian Prehierarchical Follicle Granulosa Cells
Source: Animals (Basel). 2020 Sep 6;10(9):1589. doi: 10.3390/ani10091589 (PMC7552126; doi:10.3390/ani10091589)
Supplement: Supplementary file 1 [file animals-10-01589-s001.pdf]

animals

Supplementary Materials: Effects of RAC1 on Proliferation of Hen Ovarian Prehierarchical Follicle Granulosa Cells

Thobela Louis Tyasi [1], Xue Sun [1,2], Xuesong Shan [1], Simushi Liswaniso [1], Ignatius Musenge Chimbaka [1], Ning Qin [1,2,*] and Rifu Xu [1,2,*]

1 Department of Animal Genetics, Breeding and Reproduction, College of Animal Science and Technology, Jilin Agricultural University, Changchun 130118, China; louis.tyasi@ul.ac.za (T.L.T.); xuesun1128@163.com (Xue Sun) xsshan@gmail.com (Xuesong Shan); smliswaniso@gmail.com (S.L.); ignatius.chimbaka@gmail.com (I.M.C.)

2 Joint Laboratory of Modern Agricultural Technology International Cooperation, Ministry of Education, Jilin Agricultural University, Changchun 130118, China

* Correspondence: ningqin@jlau.edu.cn (N.Q.); poultryxu@jlau.edu.cn (R.X.); Tel.: +86-150-4408-0931(N.Q.)

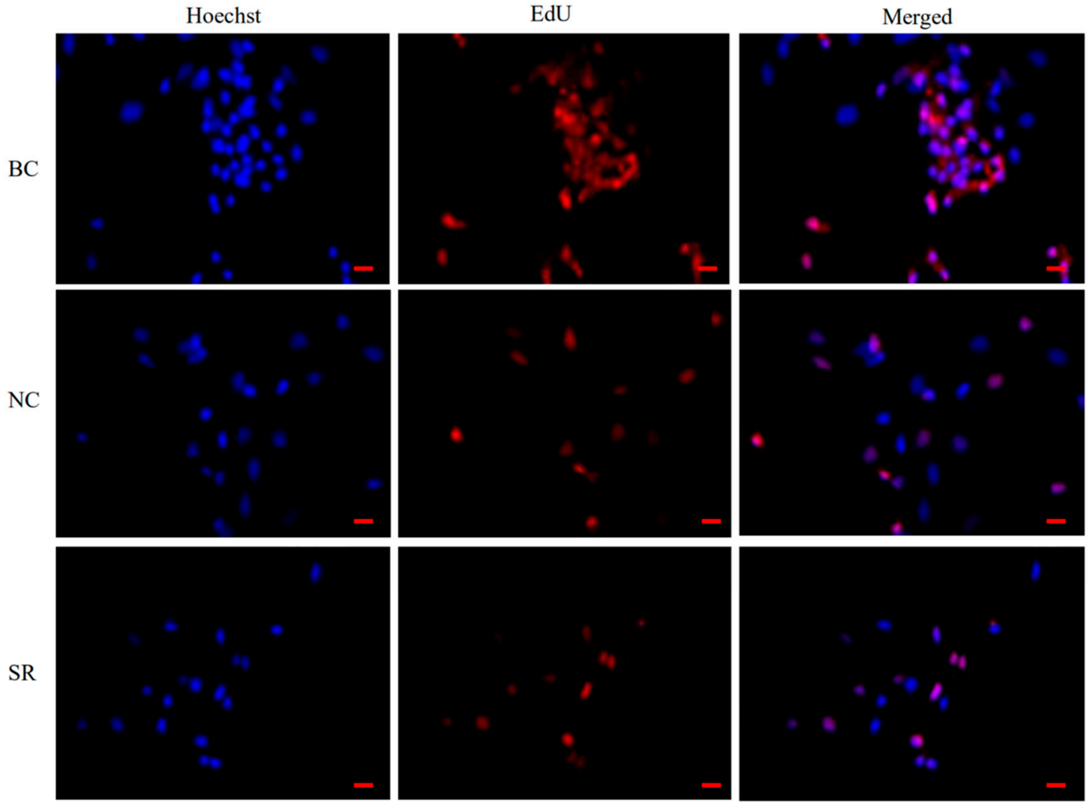

Figure S1. Effects of silencing *RAC1* on granulosa cell proliferation. The GCs were transfected with different treatment groups. SR group: exact siRNA *RAC1*; NC group: scrambled siRNA; BC group: absence of siRNA. All cell nuclei show blue fluorescence indicative of Hoechst33342 staining, the EdU-labeled cells showed red fluorescence indicating their newly synthesized DNA (original magnification ×40).

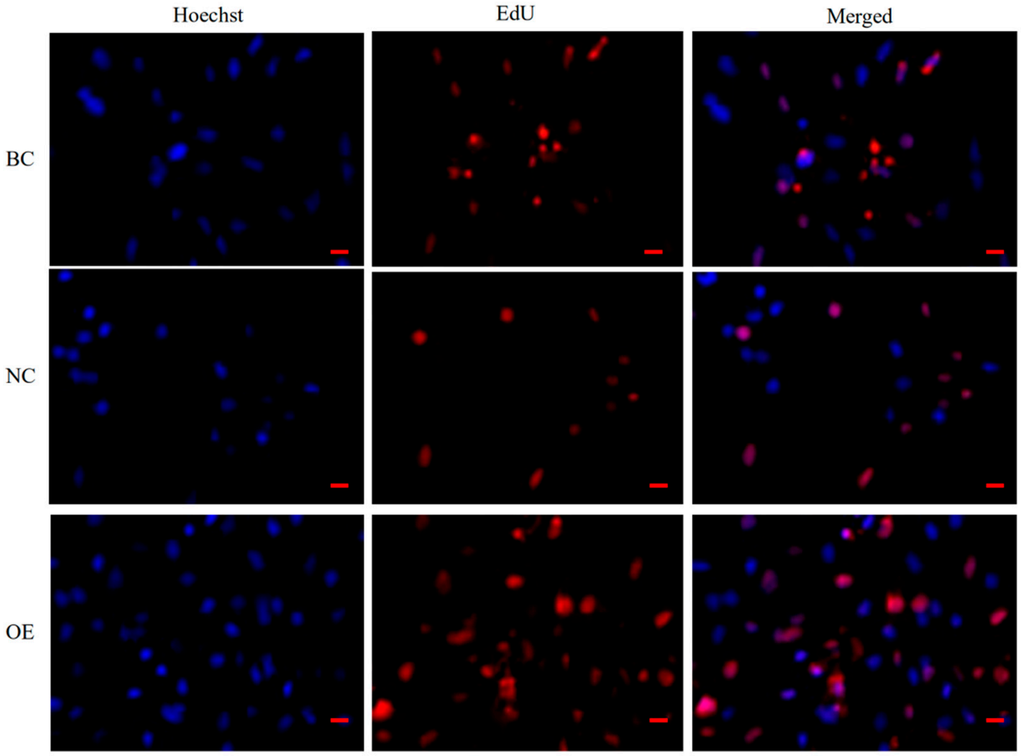

Figure S2. The effects EdU cell proliferation assay was used to assess the effects of overexpressing RAC1 on GC proliferation. The GCs were transfected with different treatment groups. OE group: pYr-adshuttle-4-RAC1 vector; NC group: pYr-adshuttle-4 empty vector; BC group: absence of expression vector. Blue fluorescence was seen in all cell nuclei since Hoechst33342 staining, cells labeled with red fluorescence suggestting their newly produced DNA (original magnification ×40).
